# Genomic risk prediction of coronary artery disease in women with breast cancer: a prospective cohort study

**DOI:** 10.1186/s13058-021-01465-0

**Published:** 2021-09-30

**Authors:** Lathan Liou, Stephen Kaptoge, Joe Dennis, Mitul Shah, Jonathan Tyrer, Michael Inouye, Douglas F. Easton, Paul D. P. Pharoah

**Affiliations:** 1 British Heart Foundation Cardiovascular Epidemiology Unit, Department of Public Health and Primary Care, University of Cambridge, Cambridge, UK; 2 Department of Public Health and Primary Care, University of Cambridge, Cambridge, UK; 3 Department of Oncology, University of Cambridge, Cambridge, UK; 4 Cambridge Baker Systems Genomics Initiative, Baker Heart and Diabetes Institute, Melbourne, VIC Australia

**Keywords:** Polygenic risk score, Breast cancer, Coronary artery disease, Coronary heart disease, Cardiovascular disease, SEARCH

## Abstract

**Background:**

Advancements in cancer therapeutics have resulted in increases in cancer-related survival; however, there is a growing clinical dilemma. The current balancing of survival benefits and future cardiotoxic harms of oncotherapies has resulted in an increased burden of cardiovascular disease in breast cancer survivors. Risk stratification may help address this clinical dilemma. This study is the first to assess the association between a coronary artery disease-specific polygenic risk score and incident coronary artery events in female breast cancer survivors.

**Methods:**

We utilized the Studies in Epidemiology and Research in Cancer Heredity prospective cohort involving 12,413 women with breast cancer with genotype information and without a baseline history of cardiovascular disease. Cause-specific hazard ratios for association of the polygenic risk score and incident coronary artery disease (CAD) were obtained using left-truncated Cox regression adjusting for age, genotype array, conventional risk factors such as smoking and body mass index, as well as other sociodemographic, lifestyle, and medical variables.

**Results:**

Over a median follow-up of 10.3 years (IQR: 16.8) years, 750 incident fatal or non-fatal coronary artery events were recorded. A 1 standard deviation higher polygenic risk score was associated with an adjusted hazard ratio of 1.33 (95% CI 1.20, 1.47) for incident CAD.

**Conclusions:**

This study provides evidence that a coronary artery disease-specific polygenic risk score can risk-stratify breast cancer survivors independently of other established cardiovascular risk factors.

**Supplementary Information:**

The online version contains supplementary material available at 10.1186/s13058-021-01465-0.

## Introduction

There were approximately 2.1 million new cases of incident female breast cancer in 2018 globally, accounting for 25% of cancer cases in women [1]. Long-term survival has improved for women over the past 30 years as advances in cancer therapy have resulted in reduced cancer-specific mortality. Consequently, mortality from other causes has become more important [2], with cardiovascular disease (CVD) being the leading cause of death in older women who survive breast cancer [3]. This is partly due to the effect of cytotoxic chemotherapies and radiotherapy which are associated with an increase in cardiovascular morbidity and mortality [4–7]. In this paper, we focus on coronary artery disease (CAD), the most common type of CVD. Particularly for long-term survivors at higher CAD risk due to risk factors unrelated to their cancer and cancer therapy, adverse effects of therapy are likely to accumulate and thus become relatively more important. It seems likely that risk factors associated with CAD in the general population will also be associated with CAD in cancer survivors, but empirical evidence is needed, particularly in those treated with chemotherapy or radiotherapy. Multiple lifestyle and environmental risk factors have well-established CAD associations including smoking, body mass index (BMI), total cholesterol, type 1 and type 2 diabetes, and hypertension [8].

Inherited genetic variation is also known to affect risk: genome-wide association studies (GWAS) have identified many common genetic variants associated with CAD, and polygenic risk scores (PRS) have been shown to provide useful CAD risk discrimination [9–11]. Polygenic risk scores are an aggregation of genomic variant information and GWAS-derived weights reflecting magnitude of association for a condition of interest [12]. The motivation behind using a PRS is based on the common variant-common disease hypothesis, where much of the genetic risk for common adult-onset diseases can be attributed to the cumulative effect of many common variants with small effect sizes rather than rare variants with large effect sizes [13]. Within research assessing clinical utility of polygenic risk scores, most of the evidence appears to come from the study of CAD. While the consensus for the clinical utility of CAD PRS is still unclear [14–17], CAD PRS is potentially poised to add accuracy to clinical risk predictions, define populations who would most benefit from statin prescriptions, and estimate lifetime risk trajectories [18]. It still remains an open question as to whether existing CAD PRS can be as predictive in non-European populations, but there has been some research that has sought to validate existing PRS in a cohort of South Asian participants [19]. There are currently no studies quantifying the performance of CAD PRS for risk prediction in breast cancer survivors and, furthermore, whether polygenic risk scores interact with oncotherapy for breast cancer. Polygenic risk scores in combination with other risk factors may be useful in identifying women with breast cancer in whom the adverse effects of treatment may outweigh the benefits. The aim of this study was to evaluate the association of a published coronary artery disease polygenic risk score [10] and incident CAD outcomes in a cohort of women with breast cancer.

## Methods

### Study cohort

The Studies in Epidemiology and Research in Cancer Heredity (SEARCH) cohort is a population-based prospective study based in the Eastern Region of England, which was served by the East Anglian Cancer registry until 2002 and the Eastern Region Cancer Intelligence Unit from 2002 to 2016. Recruitment of patients was conducted from June 1996 to December 2016. Incident breast cancer cases were all cases diagnosed under the age of 70 years from July 1996 to December 2016. Patients completed a self-administered questionnaire upon recruitment, which included questions about personal information, reproductive history, and other medical history. Tumor characteristics were obtained from the national cancer registry. Follow-up was ascertained through death registration with the most recent update provided by Public Health England on May 31st, 2020. This provides the causes of death recorded on parts 1 and 2 of the death certificate. The SEARCH dataset was restricted to female breast cancer cases who had complete genotype information (*n* = 12413) for this study. The final analytic sample contained 8946 participants after removing those of non-European ancestries (*n* = 15) and those who experienced an event before diagnosis (*n* = 3452).

Linkage of the SEARCH cohort to hospital episodes statistics (HES) data was used to identify incident CAD events. HES data comprises a record for each finished consultant episode (FCE), which is a period of care for a patient under a single consultant at a single hospital [20]. Diagnoses coded for each FCE include all diagnoses noted in the clinical record. Variables of interest included the time (years) between diagnosis and hospital admission and the ICD-10 diagnosis code. The recorded episode time, admission time, or operation time elapsed since diagnosis with breast cancer in HES was considered the time of the event. For individuals with multiple records in which CAD was one of the clinical diagnoses, the earliest time to event was used as the analytical time to event. Prevalent disease at baseline was defined as an event occurring before diagnosis (encoded as negative time) and these times were excluded from the analysis.

### Genotype data

A total of 12413 individuals from the SEARCH cohort were genotyped in two batches: batch I was genotyped on the Illumina Infinium iCOGS array (*n* = 8404) and batch II on the Illumina Infinium OncoArray (*n* = 4009). Both chips provide genome-wide coverage of common variants with 211115 SNPs on the iCOGS array [21] and 533631 SNPs on the OncoArray [22]. Genotyping QC was performed as previously described [21, 22]. Genotypes were then phased using SHAPEIT and imputed into the 1000 Genomes Project reference panel (version 3) using IMPUTE version 2 for iCOGS and OncoArray.

### Calculating PRS and quality control

The polygenic risk score (PRS) used in this study was derived by Inouye et al. and is called metaGRS (henceforth referred to as PRS), which consists of approximately 1745180 variants (a detailed description of its derivation can be found in their Additional file) [10]. The set of SNPs and their corresponding weights for PRS were taken from the Polygenic Score Catalogue, which is an open database of published polygenic risk scores [23]. The PRS was calculated as a weighted sum of all the effect alleles carried using the imputed allele dosages and the published SNP effect sizes (log relative risk). Scores for each sample individual were generated using Plink 2.0 software [24]. SNPs with imputation quality scores of less than 0.3 and ambiguous strand SNPs (A/T and G/C pairs) were excluded. Multi-allelic SNPs with only two common alleles were treated as bi-allelic. All scores were standardized to zero-mean and unit variance.

### CAD events

Incident coronary artery disease events were defined as a composite endpoint of unstable angina, myocardial infarction, or death due to complications following myocardial infarction according to the *International Statistical Classification of Diseases and Related Health Problems 10*^*th*^* Revision* (ICD-10) (Additional file 1: Table S1). This composite endpoint was chosen to maximize the number of incident cases, and no differential effects were observed between predictor variables and different definitions of incident CAD events (Additional file 1: Table S2).

### Statistical analyses

All statistical analysis was performed using R 4.0.0 [25]. We investigated the association between PRS on the composite primary endpoint of the first incident coronary event using cause-specific Cox proportional hazards regression. We identified the presence of competing risks of non-CAD death (Additional file 1: Figure S1) and thus performed Cox regression treating competing events as censored [26]. Along the same vein, cumulative incidence curves are presented instead of Kaplan–Meier curves because Kaplan–Meier curves are known to represent upward-biased incidence estimates in the presence of competing risks [26]. Time zero was date of diagnosis with patients entering the at-risk cohort at date of study enrolment (left truncation). Participants were right censored on the date of first occurrence of a CAD event, death from a cause other than coronary artery disease or last follow-up. Schoenfeld residuals for variables used in modelling and time were assessed for any significant departure from the proportional hazards assumption using the “*cox.zph*” function in the *survival* package [27]. A Wald test was performed to assess whether failure events were independent of left truncation [28]. Regression models were sequentially adjusted, first using only continuous PRS as the main exposure variable adjusted for age at diagnosis (years, continuous), genotype assay (Oncoarray, iCOGs) and eight genetic principal components (PCs), and then including sequential adjustments for conventional risk factors: BMI (kg/m [2], continuous), smoking status (never, past, current); sociodemographic variables: drinking status (past, current), education level (below GSCE, GSCE, A-level, graduate), index of multiple deprivation (IMD) (continuous); medical variables: age at menarche (years, continuous), thyroid disease (binary), parity (ordinal), hormone replacement therapy (binary); and oncotherapy variables: chemotherapy (binary), radiotherapy (binary), and hormone therapy (binary). Note that we did not have available data on baseline measurement for blood pressure, cholesterol, lipid-lowering medications, diabetes, or familial history.

The models were fit to the same subsample of cohort participants with increasingly more complete covariates to allow for more consistent comparison of the impact of adjustments and reduce the potential for selection bias in the scenario of outset restriction to participants with the most complete information on adjustment covariates.

We additionally assessed possible variation of the association of PRS with CAD according to smoking status and BMI level based on interaction tests. We also assessed the incremental improvement in CAD risk prediction from the addition of PRS to models including combinations of age, BMI, smoking, and other baseline covariates.

We calculated the net reclassification improvement and incremental discrimination index using the *ncirens* package to explore the potential clinical utility of PRS in women with breast cancer. More details about these calculations can be found in the Additional file. All confidence intervals are shown at the 95% level. All p values are 2-tailed.

## Results

### Genotyped participant characteristics

The study cohort of women with breast cancer comprised 12413 participants who had complete genotype information. The mean age at diagnosis was 54.6, and almost all participants were of European ancestries. The median [5th, 95th percentiles] time from diagnosis to entry into the study was 1.8 years [0.4, 4.3], and the median follow-up time was 10.3 years [2.6, 19.4]. A total of 750 individuals experienced a CAD event during follow-up. Out of the genotyped participants, 9496 (77%) received adjuvant hormonal therapy, 8773 (71%) received radiotherapy, and 4735 (38%) received adjuvant chemotherapy. A summary of other lifestyle, medical treatment, breast cancer, and medical history characteristics is presented in Table 1. Summary information is provided for the full SEARCH cohort (*n* = 15,755) and participants who died from breast cancer in the Additional file 1: Table S3.

**Table 1:** Genotyped participant characteristics by incident CAD outcome observed during follow-up

	SEARCH	Experienced incident CAD	Did not experience incident CAD
	*N* = *12,413*	*N* = *750*	*N* = *11,663*
**Age at diagnosis**	54.6 (8.9)	59.9 (7.1)	54.2 (8.9)
**Received adjuvant chemotherapy**	4735 (38%)	202 (27%)	4533 (39%)
**Received adjuvant radiotherapy**	8773 (71%)	535 (71%)	8238 (71%)
**Received adjuvant hormonal therapy**	9496 (77%)	580 (77%)	8916 (76%)
**Histopathological grade**	**/10859**	**/10207**	**/652**
Well Differentiated	2088 (19%)	132 (20%)	1956 (19%)
Moderately Differentiated	5364 (49%)	351 (54%)	5013 (49%)
Poorly/Undifferentiated	3407 (31%)	169 (26%)	3238 (32%)
Tumor maximum diameter (mm)*	17 [11, 24]	15.5 [10, 23]	17 [11, 24]
(Missing)	2655	156	2499
Number of nodes excised*	8 [4, 14]	8 [4,13]	8 [4, 14]
(Missing)	2934	170	2764
Number of nodes involved*	0 [0, 1]	0 [0, 1]	0 [0, 1]
(Missing)	3219	198	3021
**ER Status**	/9150	/548	/8602
Negative	1474 (16%)	80 (15%)	1394 (16%)
Positive	7676 (84%)	468 (85%)	7208 (84%)
**Highest level of education received**	**/11196**	**/666**	**/10530**
Below GSCE	2376 (21%)	238 (36%)	2138 (20%)
GSCE or similar	5721 (51%)	322 (48%)	5399 (51%)
A-level or similar	1493 (13%)	54 (8.1%)	1439 (14%)
Graduate	1606 (14%)	52 (7.8%)	1554 (15%)
**Index of Multiple Deprivation**	13.7 (9.4)	15.1 (10.1)	13.6 (9.3)
**Age at menarche**	12.8 (1.6)	12.8 (1.7)	12.8 (1.5)
(Missing)	1463	73	1390
Number of full-term pregnancies*	2 [1, 2]	2 [1, 3]	2 [1, 2]
(Missing)	5	0	5
**Height (cm)**	204 (167)	190 (138)	205 (168)
**Weight (kg)**	109 (171)	107 (157)	109 (172)
**BMI (kg/m**^**2**^**)**	26.8 (5.2)	28.6 (5.6)	26.7 (5.2)
(Missing)	830	39	791
**Received hormonal replacement therapy**	4762/11888 (40%)	381/728 (52%)	4381/11160 (39%)
**Smoking history**	**/11815**	**/719**	**/11096**
Never	6363 (54%)	305 (42%)	6058 (55%)
Past	3534 (30%)	262 (36%)	3272 (29%)
Current, in last year	1918 (16%)	152 (21%)	1766 (16%)
**Alcohol Consumption**	**/12413**	**/750**	**/11663**
Past	4290 (35%)	341 (45%)	3949 (34%)
Current	8123 (65%)	409 (55%)	7714 (66%)
**Ethnicity**	**/11980**	**/731**	**/11249**
European	11,955 (~ 100%)	730 (~ 100%)	11,225 (~ 100%)
African	1 (< 0.1%)	0	1 (< 0.1%)
Asian	2 (< 0.1%)	1 (0.1%)	1 (< 0.1%)
Southeast Asian	0	0	0
Other	22 (0.2%)	0	22 (0.2%)
**Thyroid disease**	1288/11874 (11%)	115/724 (16%)	1173/11150 (11%)

Mean (SD) is presented for continuous variables. n/N (%) is presented for categorical variables. Median [p25, p75] is presented for count variables

### Association of PRS and incident CAD

Age-adjusted models were initially assessed to understand individual associations of each variable with incident CAD survival (Table 2). There was no evidence of a departure from the proportional hazards assumptions for any of the variables modelled, and left truncation was found to be independent (Additional file 1: Table S4). There was no association between PRS and breast cancer-specific survival (HR 1.02; 95% CI 0.96–1.08).

**Table 2:** Age-adjusted univariate associations of baseline characteristics and incident CAD

Variable	Hazard Ratio (95% CI)	*P* value
PRS	1.36 (1.23, 1.50)	6.0 × 10^–10^
**Sociodemographic**		
Log(IMD)	1.25 (1.08, 1.45)	1.9 × 10^–3^
Education Level*		
GSCE or similar	0.65 (0.51, 0.82)	2.0 × 10^–4^
A-level or similar	0.60 (0.42, 0.85)	3.8 × 10^–3^
Graduate	0.50 (0.34, 0.74)	4.6 × 10^–4^
**Lifestyle**		
Log(BMI)	5.11 (3.05, 8.6)	6.1 × 10^–10^
Smoking^†^		
Past	1.44 (1.16, 1.78)	8.2 × 10^–4^
Current	1.93 (1.50, 2.51)	4.8 × 10^–7^
Drinking^††^	0.63 (0.51, 0.76)	1.6 × 10^–6^
**Medical**		
Age at menarche	0.98 (0.92, 1.05)	0.56
Parity (number of full-term pregnancies)	1.12 (1.05, 1.21)	7.6 × 10^–4^
Hormone replacement therapy	1.17 (0.96, 1.42)	0.11
Thyroid disease	1.16 (0.87, 1.54)	0.29
**Oncotherapy**		
Chemotherapy	1.02 (0.81, 1.28)	0.87
Radiotherapy	0.93 (0.75, 1.16)	0.53
Hormone therapy	0.84 (0.66, 1.07)	0.15

All univariable models were fit to the same sample. All models adjusted for age, genotype array, and 8 genetic PCs

^*^Education reference category is below GSCE

^†^Smoking reference category is never-smokers

^††^Drinking reference category is past-drinkers

**Fig. 1:**
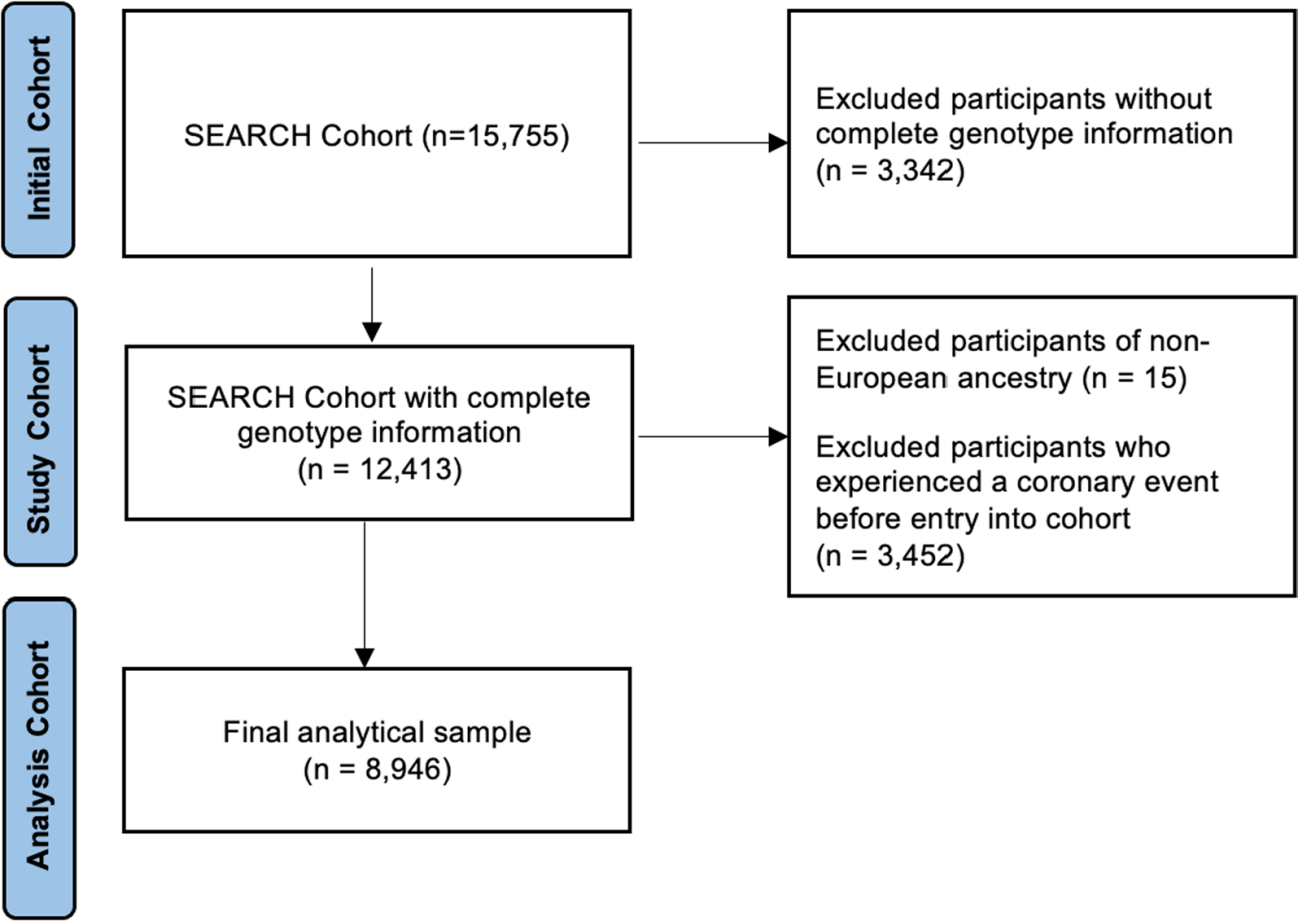
Flow diagram of selection of study cohort

The sample size for the multivariate model was 8946 with a total of 432 events after including only participants who experienced an event after entry into the study and had European ancestry (Fig. 1). The hazard ratio for incident CAD per 1 SD higher PRS adjusted only for age at diagnosis, genotype array, and eight genetic PCs was 1.36 (95% CI 1.23–1.51). Adjusting for conventional risk factors of smoking status and BMI resulted in minimal attenuation (Table 3, HR_model2_ = 1.34; 95% CI 1.21–1.49). Adjusting for other sociodemographic, lifestyle, and medical variables did not substantially change the HR of PRS (HR_model5_ = 1.33; 95% CI 1.20–1.47). A similar magnitude HR was found for another polygenic risk score, GRS49K [9] (HR_model5_ = 1.31; 95% CI 1.19–1.44). Density distributions for both standardized PRS and GRS49K can be found in Figure S2 in Additional file 1. There was no evidence that—with the inclusion or PRS and other mediator variables—chemotherapy, radiotherapy, or hormone therapy was associated with incident CAD in this study. A sensitivity analysis for follow-up only after one year, assuming a lag time to account for treatment completion within one year of diagnosis, was performed. Using model 5, the association between PRS and CAD remained largely unchanged (HR = 1.33, 95% CI 1.20–1.48).

**Table 3:** Hazard ratios for incident CAD events in sequential models adjusted for baseline mediators

Variable	Model 1 (n = 8946; d = 432)*	Model 2	Model 3	Model 4	Model 5	Model 5 *P* value	
PRS	1.36 (1.23, 1.51)	1.34 (1.21, 1.49)	1.34 (1.21, 1.48)	1.33 (1.20, 1.48)	1.33 (1.20,1.47)	7.6 × 10^–9^	
Age at diagnosis	1.10 (1.09, 1.13)	1.10 (1.09, 1.13)	1.10 (1.08, 1.12)	1.10 (1.08, 1.12)	1.10 (1.08, 1.12)	2.6 × 10^–32^	
Log(BMI)	–	4.70 (2.79, 7.92)	3.59 (2.10, 6.10)	3.59 (2.08, 6.20)	3.62 (2.09, 6.24)		3.6 × 10^–6^
Past smoker	–	1.35 (1.08, 1.67)	1.35 (1.08, 1.68)	1.33 (1.06, 1.65)	1.32 (1.06, 1.64)		0.011
Current smoker	–	1.88 (1.45, 2.45)	1.74 (1.33, 2.28)	1.70 (1.30, 2.22)	1.70 (1.30, 2.22)		9.1 × 10^–5^
GSCE education	–	–	0.73 (0.58, 0.93)	0.74 (0.58, 0.93)	0.74 (0.58, 0.94)		0.011
A-level education	–	–	0.75 (0.52, 1.07)	0.77 (0.53, 1.10)	0.77 (0.54, 1.10)		0.15
Graduate education	–	–	0.68 (0.45, 1.02)	0.69 (0.46, 1.04)	0.69 (0.46, 1.04)		0.074
Drinking	–	–	0.71 (0.57, 0.86)	0.71 (0.58, 0.87)	0.72 (0.58, 0.88)		9.3 × 10^–4^
Log(IMD)	–	–	1.09 (0.94, 1.26)	1.08 (0.93, 1.25)	1.08 (0.93, 1.25)		0.29
Age at menarche	-	–	–	0.99 (0.93, 1.06)	0.99 (0.93, 1.06)		0.86
Parity	-	–	–	1.08 (0.99, 1.16)	1.08 1.00, 1.17)		0.049
Hormone replacement therapy	-	–	–	1.20 (0.98, 1.46)	1.20 (0.98, 1.46)		0.068
Thyroid disease	-	–	–	1.02 (0.77, 1.36)	1.03 (0.77, 1.37)		0.85
Chemotherapy	–	–	–	–	0.98 (0.77, 1.24)		0.86
Radiotherapy	–	–	–	–	0.91 (0.73, 1.14)		0.42
Hormone therapy	–	–	–	–	0.87 (0.68, 1.11)		0.25

^*^All models were fit on the same sample. Model 1 represents the “baseline” model of PRS adjusted for age, genotype array and 8 genetic PCs. Model 2 is model 1 additionally adjusted for conventional risk factors of BMI and smoking. Model 3 is Model 2 additionally adjusted for other sociodemographic variables. Model 4 is Model 3 additionally adjusted for medical variables. Model 5 is Model 4 additionally adjusted for oncotherapies

### Interaction of PRS and conventional risk factors

Interactions between PRS and established cardiovascular risk factors, log(BMI) and smoking, were added separately to a model containing genotype array, eight genetic PCs, log(BMI), smoking, education level, drinking, parity, hormone replacement therapy (Table 4). The baseline mediators were selected based on multivariate results (Table 3**,** Model 5). The interaction effect between PRS and log(BMI) scaled to the mean was not significant at the 95% confidence level. Addition of an interaction term between PRS and smoking status slightly attenuated the effect of PRS, and the interaction effect between being a past smoker and the PRS approached nominal significance (*P* = 0.069). The joint hazard ratios are presented in Table 5.

**Table 4:** Hazard ratios for incident CAD events in interaction models adjusted for baseline mediators

Interaction Model	Hazard Ratio (95% CI)	*P* value
With BMI*		
PRS	1.31 (1.18, 1.46)	1.5 × 10^–7^
log(BMI)	1.24 (1.12, 1.37)	2.0 × 10^–5^
PRS*log(BMI)	1.07 (0.97, 1.18)	0.17
**With smoking**		
PRS	1.24 (1.06, 1.44)	4.0 × 10^–3^
Past smoker	1.26 (1.00, 1.58)	0.044
Current smoker	1.72 (1.31, 2.27)	9.1 × 10^–5^
PRS*past smoker	1.23 (0.98, 1.53)	0.069
PRS*current smoker	1.03 (0.79, 1.34)	0.82

Baseline mediators include age, genotype array, 8 genetic PCs, log(BMI), smoking, education level, drinking, parity, hormone replacement therapy

^*^log(BMI) was scaled to its mean

**Table 5:** Joint hazard ratios for incident CAD events in interaction models adjusted for baseline mediators

Interaction model	Joint hazard ratio (95% CI)
With BMI*	1 Unit Increase in PRS
log(BMI) (Reference)	1.31 (1,18, 1.46)
1 Unit Increase in log(BMI)	1.40 (1.24, 1.58)
**With smoking**	
Never (Reference)	1.24 (1.06, 1.44)
Past smoker	1.52 (1.29, 1.79)
Current smoker	1.27 (1.03, 1.58)

Baseline mediators include age, genotype array, 8 genetic PCs, log(BMI), smoking, education level, drinking, parity, hormone replacement therapy

^*^log(BMI) was scaled to its mean

### Interaction of PRS and oncotherapy

The hazard ratios of the interaction terms between PRS and radiotherapy, PRS and chemotherapy, and PRS and anti-hormone therapy were 1.15 (0.92, 1.43), 0.93 (0.74, 1.16), and 1.15 (0.90, 1.46) respectively in a model containing genotype array, eight genetic PCs, log(BMI), smoking, education level, drinking, parity, and hormone replacement therapy (Additional file 1: Tables S6–S7).

### Ability of PRS to risk-stratify incident CAD in breast cancer survivors

**Fig. 2:**
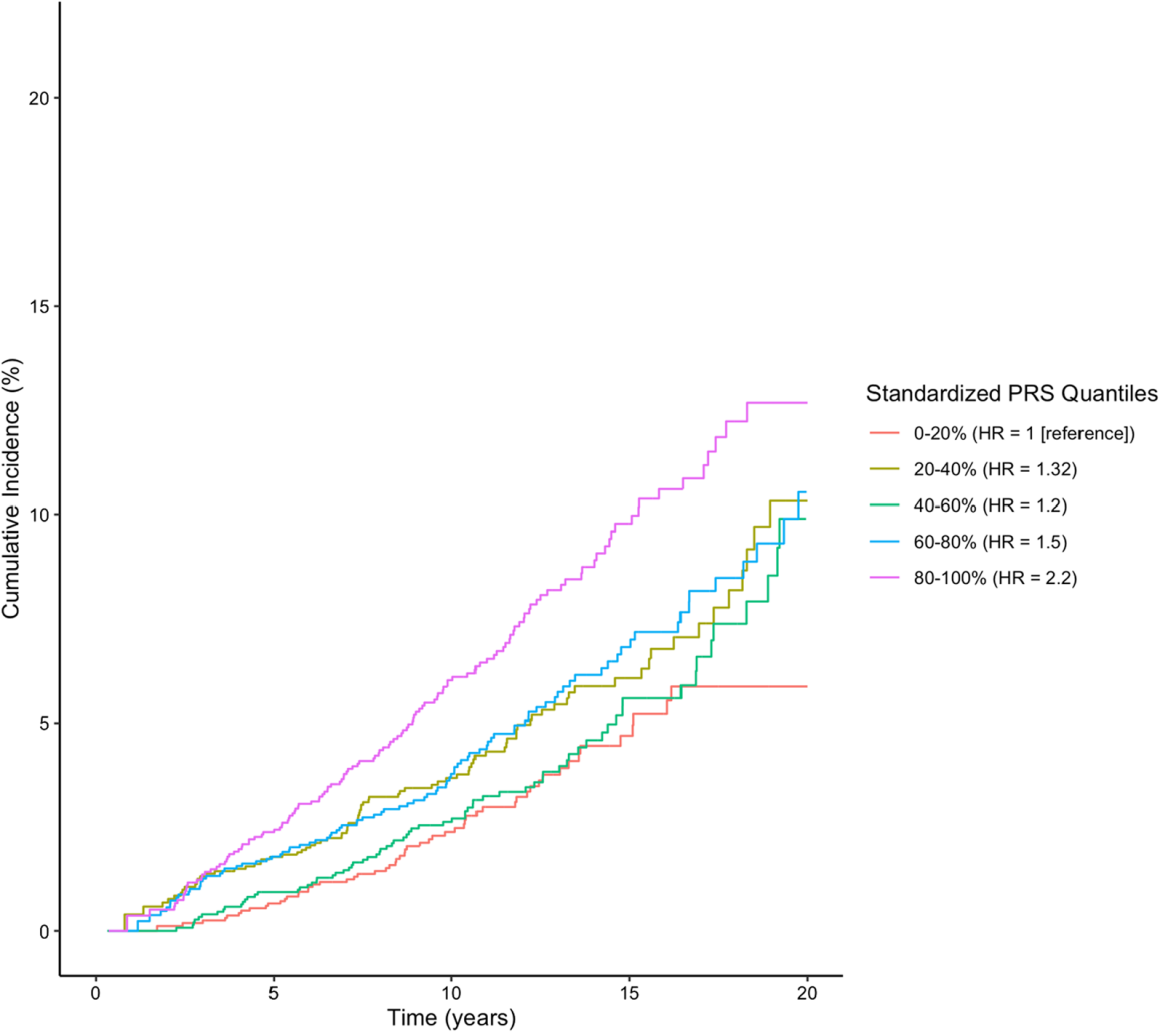
Cumulative risk of CAD by quintiles of metaGRS truncated at 20 years post-diagnosis

Figure 2 shows the cumulative risk of CAD by PRS quintile. Women in the lowest quintile of risk reached 5% cumulative incidence at 15.1 years compared to 8.9 years for women in the highest quintile of risk.

**Fig. 3:**
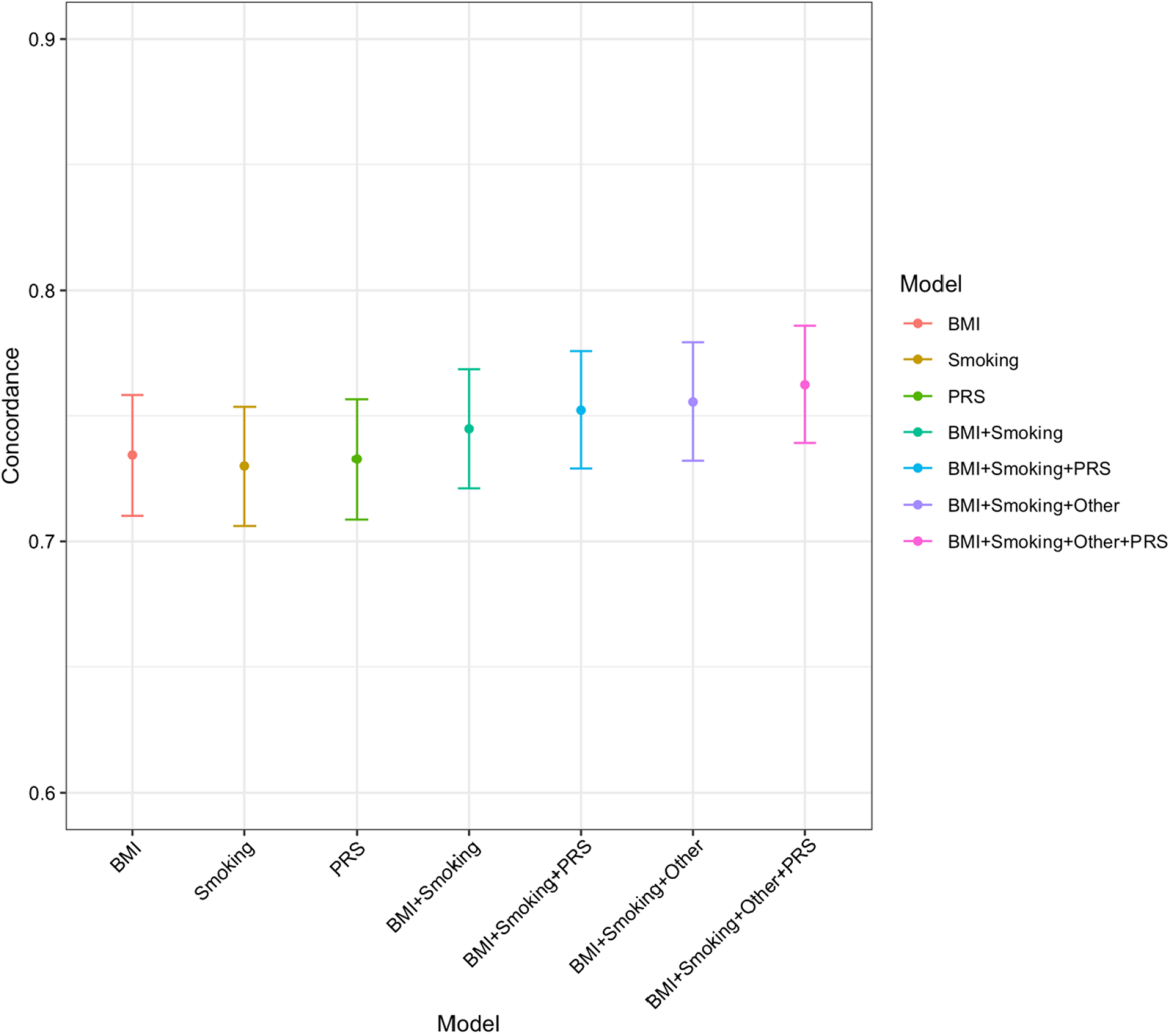
Training C-indices for conventional risk factors and metaGRS

The discrimination of the PRS alone measured by the c-index was somewhat less than that of a model with BMI and smoking combined (0.73 vs. 0.74, *P* = 0.048) after adjusting for age, genotype array, and genetic PCs (Fig. 3). The addition of PRS made little practical improvement to a model including genotype array, eight genetic PCs, BMI, smoking status, education level, drinking, IMD, age at menarche, parity, hormone replacement therapy, and thyroid disease (0.757 vs. 0.764, *P* = 0.052). The total proportion of CAD and non-CAD cases that was reclassified was 22% and 11% respectively. The net proportion of CAD and non-CAD cases assigned to a higher risk category was 12% and 5.6% respectively for a 10-year CAD incidence risk (Additional file 1: Table S8).

## Discussion

Based on a large cohort of British women with breast cancer, we have provided evidence that a CAD polygenic risk score developed for the general population can be generalized to breast cancer patients, with an estimated 33% higher CAD risk per 1 SD higher PRS (HR = 1.33, 95% CI 1.20, 1.47), independent of established cardiovascular risk factors (age, smoking, BMI), oncotherapies and other variables associated with cardiovascular risk (education level) in a cohort of British women with breast cancer. Our results support previous evidence that the association of PRS and CAD risk may operate through molecular pathways that do not overlap with those of traditional risk factors such as smoking and BMI. This is consistent with the original PRS analysis which found only a modest attenuation for PRS when adjusting for BMI, smoking status, as well as diabetes, hypertension, family history of heart disease, and cholesterol levels (HR: 1.58 per SD; 95% CI 1.55–1.61 unadjusted; HR: 1.48 per SD; 95% CI 1.45–1.51 adjusted) [10]. Several other studies also found only modest attenuation of CAD polygenic risk scores when adjusting for variables such as lipid treatment at baseline, cholesterol, and systolic blood pressure [9, 19, 29].

However, we note that there was an almost significant interactive effect between being a past smoker and the PRS. Since PRS is known to be correlated to certain conventional CAD risk factors [10, 30], it is plausible that some fraction of incident CAD risk explained by PRS may be dependent on smoking status. While this paper is not expressly predictive in nature, we note that the addition of PRS may not have provided additional risk discrimination on top of BMI and smoking because by middle age, the genes that compose this risk score may have already exerted their influence, and thus the PRS would not be expected to add discriminatory ability.

The PRS improves risk discrimination in breast cancer survivors. For instance, we found an over twofold HR for CAD in a comparison of individuals in the top versus bottom one-fifth of the risk score distribution. Furthermore, when considering 10-year risk of incident CAD following breast cancer diagnosis, we found that 5.6% of lower risk participants who did not have a recorded CAD event were reclassified to a higher risk group with the addition of PRS to the baseline model (Additional file 1: Table S5). While the change is discrimination is small, this may result in meaningful risk reclassification in clinical decision-making between the harms and benefits of chemotherapy. Further work is required to evaluate whether such reclassification would justify the additional cost of genotyping.

We acknowledge the limitation of how treatment (chemotherapy, radiotherapy, and anti-hormone therapy) was coded as dichotomous variable (whether or not a patient received treatment). The loss of information about other treatment aspects (e.g. dose, duration, type) may have contributed to measurement error that resulted in the associations reported in our paper. Furthermore, the association of the interaction of PRS and chemotherapy is likely explained by selection bias, where healthy patients are more likely to undergo chemotherapy. More granular data will be required to further assess these associations.

The role that PRS may play in breast cancer clinical care is currently unclear, but fundamentally, PRS may be used to help estimate the lifetime risk of cardiovascular disease in a breast cancer survivor. This may have two clinically useful benefits: (1) facilitate earlier detection of cardiovascular risk in breast cancer survivors to help them more effectively manage cardiovascular risk factors earlier to reduce future cardiovascular risk and (2) aid in treatment decision-making when considering the negative cardiotoxic effects of their treatment regiments.

This is especially important in breast cancer patients who face the unique challenge of needing to maximize gains from cancer treatment while also minimizing its cardiotoxic effects. More women are surviving breast cancer with an increase in 5-year survival for early stage breast cancer from 79% in 1990 to 88% in 2012 [31] (there were an estimated 3.4 million breast cancer survivors in the US in 2015 [32]), so cardiovascular mortality may become an increasingly important concern. Bradshaw et al. showed that there is nearly a twofold increase in the incidence of CVD for long-term breast cancer survivors around 7 years after diagnosis [4]. Several large randomized trials have provided evidence of the association between chemotherapy, radiotherapy, hormone therapy and increased risk of cardiovascular events [33, 34]. For instance, Darby et al. showed that the rate of CAD was proportional to the average dose of ionizing radiation during radiotherapy for breast cancer, with increases in rate continuing as long as 20 years post-exposure [35]. This is particularly important for women diagnosed at a relatively young age who begin treatment and may then have increased risk of CVD mortality. It suggests that breast cancer survivors may benefit from a PRS assessment and should be closely monitored for development of cardiovascular risk factors following diagnosis and subsequent treatment. In our study, cumulative incidence curves of incident CAD events did not appear to be substantially different when stratified by oncotherapy status (Additional file 1: Figure S3), which suggests that more granular data on treatment data, such as dosage, frequency, or duration, is needed to better assess the interplay between drug cardiotoxicity and genetic cardiovascular susceptibility. PRS may help clinicians and their patients make decisions about whether the benefits of adjuvant chemotherapy and other oncotherapies outweigh the risks.

### Limitations

There are some limitations in interpreting the current findings. The association between PRS and incident CAD could not be adjusted for important risk factors such as diabetes, hyperlipidaemia, family history of cardiovascular disease, and hypertension because these data were not collected. It is worth noting; however, that the PRS used in this study has been shown in other cohorts to provide additional predictive benefit over standard cardiovascular risk prediction algorithms such as the Framingham risk score, which include such metabolic risk factors [9]. Treatment data were limited to whether the patient had received chemotherapy, radiotherapy, and anti-hormone therapy. Data on specific drugs or doses received were not available. Genotype data were available for predominantly participants of white European ancestry, which suggests the need for studies in people of other ancestries to maximize generalizability. Furthermore, the observational nature of these data limits any inference that might be drawn relating to the association between therapy and outcome.

## Conclusion

Cardiovascular disease is an important long-term risk among women who survive breast cancer. This risk is increased by some breast cancer therapies. Comprehensive risk models for cardiovascular disease have the potential to help in the clinical management of this risk and may improve long-term outcomes for these women.

## Supplementary Information

**Additional file 1**. Supplementary appendix containing additional description of methods and tables and figures for sensitivity analyses described in the main text.

## Data Availability

The data are available from the authors on request. The HES data were supplied by PHE, and they will not give permission for the data to be shared.
