# Supplementary material for: Genomic risk prediction of coronary artery disease in women with breast cancer: a prospective cohort study
Source: Breast Cancer Res. 2021 Sep 30;23:94. doi: 10.1186/s13058-021-01465-0 (PMC8482562; doi:10.1186/s13058-021-01465-0)
Supplement: Supplementary file 1 — Additional file 1. Supplementary appendix containing additional description of methods and tables and figures for sensitivity analyses described in the main text. [file 13058_2021_1465_MOESM1_ESM.docx]

**SUPPLEMENTARY APPENDIX**

**Supplementary Methods**

**Calculation of Net Reclassification Improvement (NRI)**

The initial motivation behind NRI was to evaluate the prediction increment of a new predictor other than using the change in AUC. Kerr et. al. 2015 recommends that NRIs should be reported separately for events (cases) and nonevents (controls).

$${NRI}_{e}=P\left( up | event \right)-P\left( down | event \right)$$

$${NRI}_{ne}=P\left( down | nonevent \right)-P\left( up | nonevent \right)$$

where “up” means that the new risk model places a person into a higher risk category than the old model, and “down” means the new model places a person into a lower risk category. A reclassification table indicates the number of individuals who move to another risk category or remain in the same risk category as a result of updating the risk model. The base risk model included array type, age, log(BMI), smoking, education level, alcohol, log(IMD), age at menarche, parity, thyroid disease, and hormone replacement therapy. The updated risk model added PRS to the base model. Categorical NRI equal to x% means that compared with individuals without outcome, individuals with outcome were x% more likely to move up a category than down. Continuous NRI, which is a popular metric, was not included because it has no interpretation that translates to clinical benefit for several reasons that are explained in Kerr et. al. 2015. Confidence intervals were constructed using a bootstrap estimate of the variance of NRI using the *ncirens* R package.

**Calculation of Incremental Discrimination Index**

The Incremental Discrimination Index (IDI) was also calculated as:

$$IDI=P_{new}\left( case \right)-P_{new}\left( noncase \right)-P_{old}\left( case \right)+P_{old}(noncase)$$

An IDI equal to x% means that the difference in average predicted risks between the individuals with and without the outcome increased by x% in the updated model.

## Supplementary Tables

**Table S1.** Incident CAD Outcome Definitions

Incident CAD outcome definitions

| **Outcome** | **ICD-10** |
| --- | --- |
| Acute myocardial infarction | I21, I22 |
| Death due to complications following myocardial infarction | I23 |
| Death due to other CAD outcomes | I24, I25 |

**Table S2.** Age-adjusted hazard ratios for 1 SD increase in metaGRS for different CAD definitions

| **Definition** | **Events** | **HR** | **95% CI** |
| --- | --- | --- | --- |
| General (I20-25) | 571 | 2.08 | (1.72, 2.51) |
| Clinical (I21-25) | 522 | 2.09 | (1.72, 2.55) |
| MI (I21-23) | 126 | 2.57 | (1.72, 3.84) |
| Other (I24-25) | 503 | 2.07 | (1.69, 2.53) |

*Restricted to European ancestry participants who experience an incident event only after entry into the study

**Table S3.** Participants by breast cancer outcome observed during follow-up

|  | **SEARCH** | **Died from breast cancer** | **Breast cancer survivor** |
| --- | --- | --- | --- |
|  | ***N=15,755*** | ***N=2,217*** | ***N=13,538*** |
| **Age at diagnosis** | 54.1 (9.1) | 52.1 (9.6) | 54.4 (8.9) |
| **Received adjuvant chemotherapy** | 5927 (38%) | 1440 (65%) | 4487 (33%) |
| **Received adjuvant radiotherapy** | 10753 (68%) | 1702 (77%) | 9051 (67%) |
| **Received adjuvant hormonal therapy** | 11801 (75%) | 1662 (75%) | 10139 (75%) |
| **Histopathological grade** | **/13282** | **/1856** | **/11426** |
| Well Differentiated | 2553 (19%) | 131 (7%) | 2422 (21%) |
| Moderately Differentiated | 6466 (49%) | 848 (46%) | 5618 (49%) |
| Poorly/Undifferentiated | 4263 (32%) | 877 (47%) | 3386 (30%) |
| **Tumour maximum diameter (mm)*** | 17 [11, 25] | 24 [18, 35] | 16 [11, 23] |
| (Missing) | 4459 | 904 | 3555 |
| **Number of nodes excised*** | 8 [4, 14] | 11 [7, 16] | 8 [4, 14] |
| (Missing) | 4810 | 941 | 3869 |
| **Number of nodes involved*** | 0 [0, 1] | 2 [0, 5] | 0 [0, 1] |
| (Missing) | 5126 | 959 | 4167 |
| **ER Status** | **/11301** | **/1596** | **/9435** |
| Negative | 1855 (17%) | 406 (25%) | 1449 (15%) |
| Positive | 9176 (83%) | 1190 (75%) | 7986 (85%) |
| **Highest level of education received** | **/14217** | **/2038** | **/12179** |
| Below GSCE | 2684 (19%) | 293 (14%) | 2391 (20%) |
| GSCE or similar | 7641 (54%) | 1233 (61%) | 6408 (53%) |
| A-level or similar | 1896 (13%) | 249 (12%) | 1647 (14%) |
| Graduate | 1996 (14%) | 263 (13%) | 1733 (14%) |
| **Index of Multiple Deprivation** | 13.8 (9.4) | 14.5 (9.9) | 13.7 (9.4) |
| **Age at menarche** | 12.8 (1.6) | 12.8 (1.6) | 12.8 (1.6) |
| (Missing) | 2040 | 218 | 1822 |
| **Number of full-term pregnancies*** | 2 [1, 2] | 2 [1, 2] | 2 [1, 2] |
| (Missing) | 7 | 0 | 7 |
| **Height (cm)** | 207 (173) | 197 (153) | 209 (176) |
| **Weight (kg)** | 114 (181) | 107 (165) | 115 (184) |
| **BMI (kg/m^2^)** | 26.7 (5.1) | 27.0 (5.1) | 26.7 (5.1) |
| (Missing) | 1139 | 129 | 1010 |
| **Received hormonal replacement therapy** | 5720/14779 (39%) | 709/2116 (34%) | 5011/12663 (40%) |
| **Smoking history** | **/14928** | **/2124** | **/12804** |
| Never | 8096 (54%) | 1175 (55%) | 6921 (54%) |
| Past | 4395 (29%) | 589 (28%) | 3806 (30%) |
| Current, in last year | 2437 (16%) | 360 (17%) | 2077 (16%) |
| **Alcohol Consumption** | **/15755** | **/2217** | **/13538** |
| Past | 5614 (36%) | 832 (38%) | 4782 (35%) |
| Current | 10141 (64%) | 1385 (62%) | 8756 (65%) |
| **Ethnicity** | **/15126** | **/2145** | **/12981** |
| European | 14901 (99%) | 2113 (99%) | 12788 (99%) |
| African | 49 (0.3%) | 7 (0.3%) | 42 (0.3%) |
| Asian | 66 (0.4%) | 11 (0.5%) | 55 (0.4%) |
| Southeast Asian | 29 (0.2%) | 4 (0.2%) | 25 (0.2%) |
| Other | 81 (0.5%) | 10 (0.5%) | 71 (0.5%) |
| **Thyroid disease** | 1553/14992 (10%) | 201/2125 (10%) | 1352/12867 (11%) |

Summary statistics presented are mean (SD) or n/N (%) or median [p25, p75]

**Table S4.** Test for violation of assumption of proportional hazards

| **Variable** | **P-value** |
| --- | --- |
| DiagnosisAge | 0.3541756 |
| Chemotherapy | 0.5498367 |
| Radiotherapy | 0.1532279 |
| HormoneTherapy | 0.3982543 |
| eduCat | 0.1336767 |
| AgeMenarche | 0.0692628 |
| Parity | 0.4802935 |
| height | 0.2569031 |
| weight | 0.0821325 |
| BMI | 0.0277517 |
| HRTEver | 0.6330726 |
| smokingEver | 0.6465531 |
| AlcNow | 0.0499855 |
| EthnicityClass | 0.7334602 |
| IMD | 0.0309320 |
| prs_z | 0.5411762 |
| metagrs_z | 0.4382610 |
| GLOBAL | 0.3015880 |

Left truncation is independent from time of entry into study following diagnosis (Wald test p-value = 0.13)

## Table S5. Hazard ratios for incident CAD events for metaGRS deciles

| **PRS Decile*** | **HR (95% CI)*** | **P-value** |
| --- | --- | --- |
| **2** | 0.99  (0.58, 1.68) | 0.97 |
| **3** | 1.09  (0.65, 1.80) | 0.74 |
| **4** | 1.55  (0.97, 2.48) | 0.067 |
| **5** | 1.00  (0.60, 1.68) | 0.99 |
| **6** | 1.38  (0.85, 2.23) | 0.19 |
| **7** | 1.54  (0.96, 2.47) | 0.073 |
| **8** | 1.46  (0.91, 2.34) | 0.12 |
| **9** | 1.68  (1.05, 2.68) | 0.03 |
| **10** | 2.71  (1.75, 4.19) | 6.8x10^-6^ |

*The lowest decile was the reference category.

**Table S6.** Hazard ratios for incident CAD events in interaction models adjusted for baseline mediators

| **Interaction Model** | **Hazard Ratio (95% CI)** | ***P* value** |
| --- | --- | --- |
| **With Radiotherapy** |  |  |
| PRS | 1.21 (1.01, 1.45) | 0.035 |
| Radiotherapy | 0.87 (0.70, 1.08) | 0.21 |
| PRS*Radiotherapy | 1.15 (0.92, 1.43) | 0.20 |
| **With Chemotherapy** |  |  |
| PRS | 1.36 (1.21, 1.53) | 1.1x10^-7^ |
| Chemotherapy | 1.01 (0.79, 1.27) | 0.95 |
| PRS*Chemotherapy | 0.93 (0.74, 1.16) | 0.50 |
|  |  |  |
| **With Anti-Hormone Therapy** |  |  |
| PRS | 1.20 (0.97, 1.48) | 0.089 |
| Anti-Hormone Therapy | 0.84 (0.65, 1.07) | 0.15 |
| PRS*Anti-Hormone Therapy | 1.15 (0.90, 1.46) | 0.26 |

Baseline mediators include age, genotype array, 8 genetic PCs, log(BMI), smoking, education level, drinking, parity, hormone replacement therapy

**Table S7.** Joint hazard ratios for incident CAD events in interaction models adjusted for baseline mediators

| **Interaction Model** | **Joint Hazard Ratio (95% CI)** |
| --- | --- |
| **With Radiotherapy** | 1 Unit Increase in PRS |
| No Radiotherapy (Reference) | 1.21 (1.01, 1.45) |
| Radiotherapy | 1.39 (1.24, 1.56) |
| **With Chemotherapy** |  |
| No Chemotherapy (Reference) | 1.36 (1.21, 1.53) |
| Chemotherapy | 1.27 (1.05, 1.52) |
|  |  |
| **With Anti-Hormone Therapy** |  |
| No Anti-Hormone Therapy (Reference) | 1.20 (0.97, 1.48) |
| Anti-Hormone Therapy | 1.37 (1.23, 1.53) |

## Net Reclassification Improvement

Using commonly recommended thresholds for initiation of statin therapy (10% 10-year risk)^19^, we assessed whether addition of PRS improved individual 10-year risk reclassification when added to a base model including genotype array, eight genetic PCs, age, BMI, smoking status, drinking, IMD, age at menarche, parity, thyroid disease, hormone replacement therapy. The net reclassification improvement (NRI) results are presented in Table S1. This is the retrospective NRI using a logistic model for prediction of CAD event at 10 years. In the cohort of cases, those who experienced a CAD event, 14% of lower risk (0-5%) participants were reclassified to an intermediate (5-10%) 10-year risk group. The Integrated Discrimination Index score showed an increase of 0.008 when PRS was added to the base model.

**Table S8.** Reclassification of incident CAD event risk within 10 years

|  |  | **Base Model + PRS** | | | | |
| --- | --- | --- | --- | --- | --- | --- |
|  |  | **0-5%** | **5-10%** | **10-100%** | **Total** | **Reclass %** |
| All individuals | | | | | | |
| Base Model | 0-5% | 6172 | 305 | 1 | 6478 | 5% |
|  | 5-10% | 353 | 1141 | 227 | 1721 | 34% |
|  | 10-100% | 0 | 186 | 741 | 927 | 20% |
|  | Total | 6525 | 1632 | 969 | 9126 | 12% |
| Incident CAD Present | | | | | | |
| Base Model | 0-5% | 94 | 19 | 0 | 113 | 17% |
|  | 5-10% | 18 | 81 | 27 | 126 | 36% |
|  | 10-100% | 0 | 21 | 124 | 145 | 14% |
|  | Total | 112 | 121 | 151 | 384 | 22% |
| Incident CAD Absent | | | | | | |
| Base Model | 0-5% | 6078 | 286 | 1 | 6365 | 5% |
|  | 5-10% | 335 | 1060 | 200 | 1595 | 34% |
|  | 10-100% | 0 | 165 | 617 | 782 | 21% |
|  | Total | 6413 | 1511 | 818 | 8742 | 11% |
| NRI (categorical)  [95% CI] | | NRI for events: 0.020 [-0.028-0.068]  NRI for non-events: 0.26 [0.16-0.37] | | | | |
| IDI (continuous)  [95% CI] | | 0.008 [0.004-0.011] | | | | |

**Supplementary Figures**

**Figure S1.** Cumulative incidence curves for competing risks of breast cancer and incident CAD

**
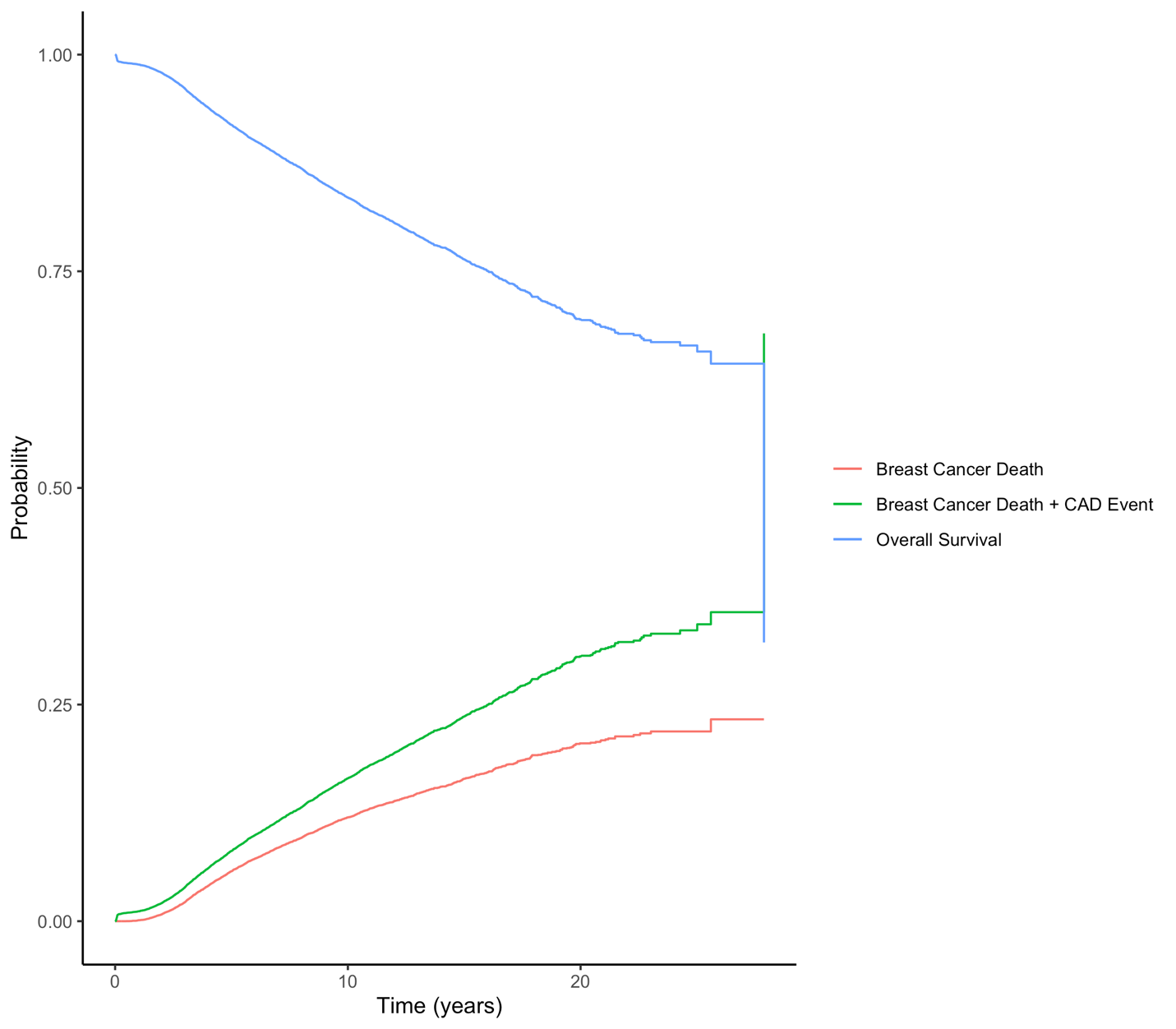
**

**Figure S2.** Distribution of the normalised PRS by incident CAD event. Vertical lines represent the mean PRS. (*top: GRS49K, bottom: metaGRS)*

**
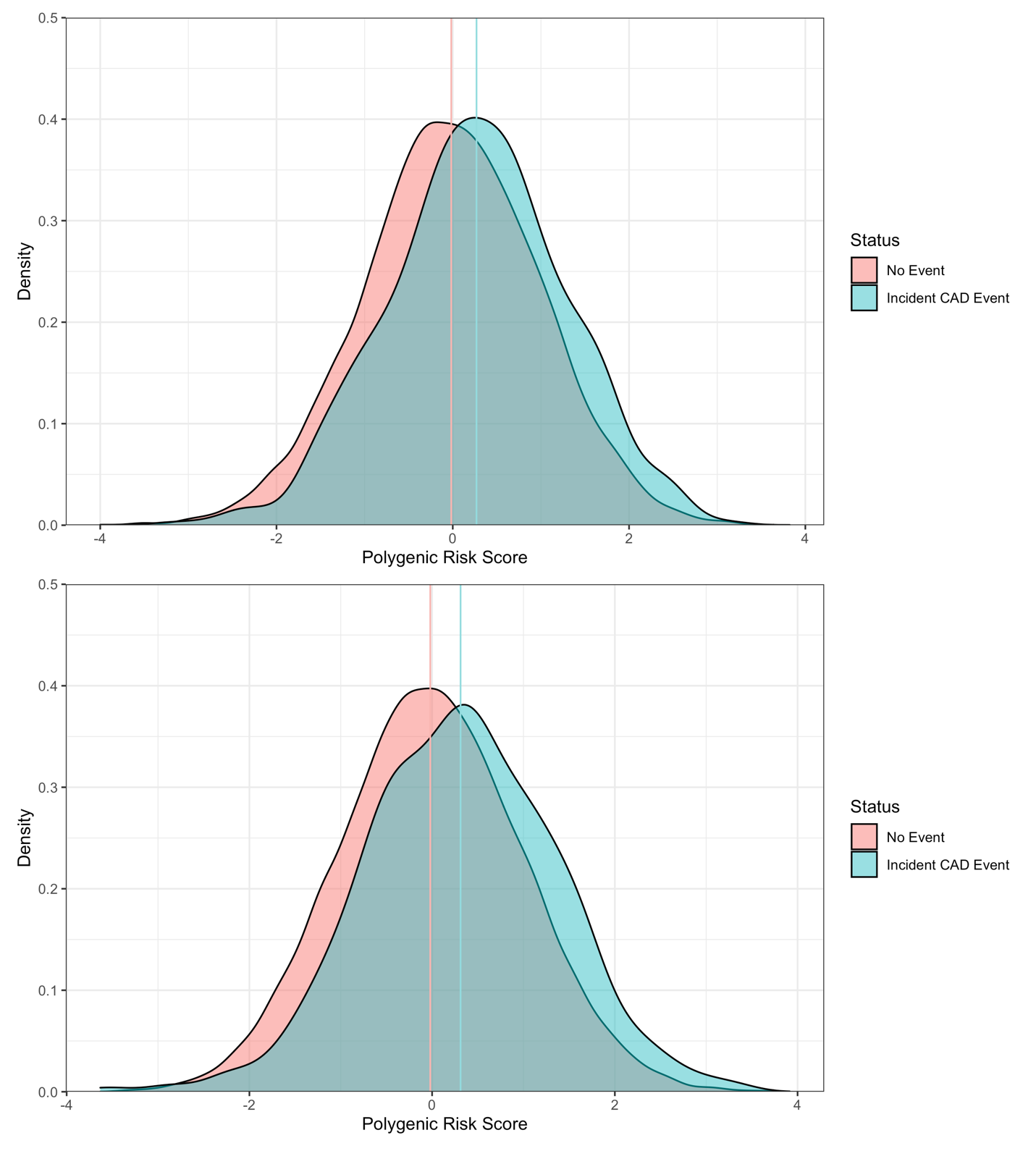
**

**Figure S3.** Cumulative incidence curves for breast cancer death and incident CAD stratified by oncotherapy

**
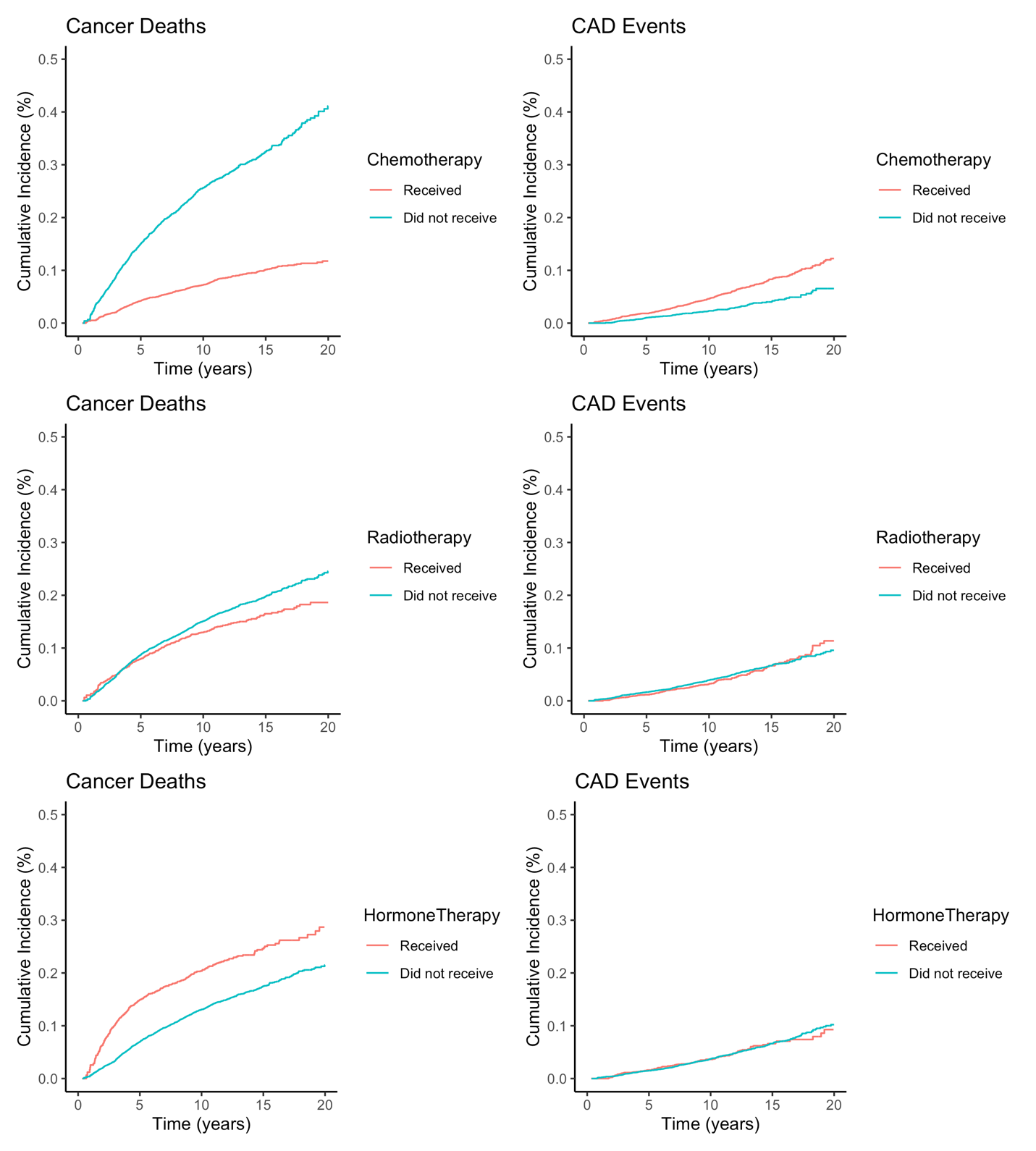
**

**References**

Kerr KF, Wang Z, Janes H, McClelland RL, Psaty BM, Pepe MS. Net reclassification indices for evaluating risk prediction instruments: a critical review. Epidemiology. 2014;25(1):114-121. doi:10.1097/EDE.0000000000000018
